# Supplementary material for: The genome sequence of the Large Nutmeg, Apamea anceps (Denis & Schiffermüller, 1775)
Source: Wellcome Open Res. 2024 Feb 19;9:61. [Version 1] doi: 10.12688/wellcomeopenres.20681.1 (PMC12501590; doi:10.12688/wellcomeopenres.20681.1)
Supplement: Peer review history

# Reviewer report: Reviewer response for version 1

**DOI:** 10.21956/wellcomeopenres.22886.r134069

**Published:** 2025-10-08

**Authors:** M. Srinivasulu Reddy

**Affiliations:** 1 Sri Venkateswara University, Tirupati, India

**Overall Evaluation**

 The manuscript provides a comprehensive and high-quality chromosomal-level genome assembly of *Apamea anceps*, representing an important contribution to lepidopteran genomics and pest management research. The experimental workflow, data acquisition, and bioinformatics pipelines are sound and consistent with established standards under the Darwin Tree of Life project. The data generated are openly available and will be a valuable reference for future comparative genomics, phylogenetics, and molecular ecology studies in *Noctuidae*.

**Technical Soundness**

- **Rationale:** Clearly described and justified.
- **Methodology:** Technically sound with sufficient detail for reproducibility.
- **Data Accessibility:** Fully compliant with open-access standards; datasets are well curated and linked.

**Overall Quality:** Excellent data note

 The manuscript represents a valuable genomic resource and is methodologically robust.

Are sufficient details of methods and materials provided to allow replication by others?

Yes

Is the rationale for creating the dataset(s) clearly described?

Yes

Are the datasets clearly presented in a useable and accessible format?

Yes

Are the protocols appropriate and is the work technically sound?

Yes

Reviewer Expertise:

Biotechnology

I confirm that I have read this submission and believe that I have an appropriate level of expertise to confirm that it is of an acceptable scientific standard.

# Reviewer report: Reviewer response for version 1

**DOI:** 10.21956/wellcomeopenres.22886.r134067

**Published:** 2025-10-06

**Authors:** Omar Hamarsheh

**Affiliations:** 1 Al-Quds University, Jerusalem, Palestinian Territory

The manuscript provides a high-quality genome assembly of *Apamea anceps* .  The writing is clear, with appropriate references and details on methodology. However, there are areas where clarification, conciseness, and interpretation could be improved to make the report more accessible and scientifically stronger.

 1- Abstract is short need to expand it

 2- genomic knowledge gaps in *Apamea* is missing

 3- compare this assembly  to other *Noctuidae* genomes

 4- give more information about insecticide resistance genes, host–plant adaptation, or population genomics potential?

Are sufficient details of methods and materials provided to allow replication by others?

Yes

Is the rationale for creating the dataset(s) clearly described?

Yes

Are the datasets clearly presented in a useable and accessible format?

Yes

Are the protocols appropriate and is the work technically sound?

Yes

Reviewer Expertise:

Molecular parasitology / infectious diseases

I confirm that I have read this submission and believe that I have an appropriate level of expertise to confirm that it is of an acceptable scientific standard.

# Reviewer report: Reviewer response for version 1

**DOI:** 10.21956/wellcomeopenres.22886.r107098

**Published:** 2024-10-28

**Authors:** Romain Villoutreix, Baptiste Pierre

**Affiliations:** 1 INRAE, Universite de Montpellier (Ringgold ID: 27037), Montpellier, Occitanie, France; 2 Universite de Montpellier (Ringgold ID: 27037), INRAE, Montpellier, Occitanie, France

The manuscript titled “The Genome Sequence of the Large Nutmeg, Apamea anceps (Denis & Schiffermüller, 1775)” presents a high-quality genome assembly for the large Nutmeg, *Apamea anceps*. The authors employed a combination of robust methodological frameworks, including third-generation long-read sequencing technology, Hi-C, and rigorous data curation.

 Significant attention was devoted to the assembly process. The authors employed advanced pipelines to create a high-quality chromosomal-level assembly. However, they could consider adapting their pipeline with more recent versions of the tools used (example : Hifiasm 0.16.1-r375, sep 2021 → 0.20.0-r639, oct 2024).

 This resource will greatly benefit genomic research in this species. I therefore recommend this article.

Are sufficient details of methods and materials provided to allow replication by others?

Yes

Is the rationale for creating the dataset(s) clearly described?

Yes

Are the datasets clearly presented in a useable and accessible format?

Yes

Are the protocols appropriate and is the work technically sound?

Yes

Reviewer Expertise:

Evolutionary Genomics

We confirm that we have read this submission and believe that we have an appropriate level of expertise to confirm that it is of an acceptable scientific standard.
